# The Making of a Productivity Hotspot in the Coastal Ocean

**DOI:** 10.1371/journal.pone.0027874

**Published:** 2011-11-21

**Authors:** Dana K. Wingfield, S. Hoyt Peckham, David G. Foley, Daniel M. Palacios, Bertha E. Lavaniegos, Reginaldo Durazo, Wallace J. Nichols, Donald A. Croll, Steven J. Bograd

**Affiliations:** 1 Institute of Marine Sciences, University of California Santa Cruz, Santa Cruz, California, United States of America; 2 Grupo Tortuguero, A.C / The Ocean Foundation, La Paz, Baja California Sur, México; 3 Center for Marine Conservation, Duke University, Beaufort, North Carolina, United States of America; 4 Environmental Research Division, Southwest Fisheries Science Center, NOAA Fisheries, Pacific Grove, California, United States of America; 5 Joint Institute for Marine and Atmospheric Research, University of Hawai'i at Manoa, Honolulu, Hawai'i, United States of America; 6 Departamento de Oceanografía Biológica, Centro de Investigación Cientifica y de Educación Superior de Ensenada, Ensenada, Baja California, Mexico; 7 Facultad de Ciencias Marinas, Universidad Autonoma de Baja California Mexico, Ensenada, Baja California, Mexico; 8 California Academy of Sciences, San Francisco, California, United States of America; 9 Ocean Revolution, Davenport, California, United States of America; 10 Department of Ecology and Evolutionary Biology, University of California Santa Cruz, Santa Cruz, California, United States of America; Phillip Island Nature Parks, Australia

## Abstract

**Background:**

Highly productive hotspots in the ocean often occur where complex physical forcing mechanisms lead to aggregation of primary and secondary producers. Understanding how hotspots persist, however, requires combining knowledge of the spatio-temporal linkages between geomorphology, physical forcing, and biological responses with the physiological requirements and movement of top predators.

**Methodology/Principal Findings:**

Here we integrate remotely sensed oceanography, ship surveys, and satellite telemetry to show how local geomorphology interacts with physical forcing to create a region with locally enhanced upwelling and an adjacent upwelling shadow that promotes retentive circulation, enhanced year-round primary production, and prey aggregation. These conditions provide an area within the upwelling shadow where physiologically optimal water temperatures can be found adjacent to a region of enhanced prey availability, resulting in a foraging hotspot for loggerhead sea turtles (*Caretta caretta*) off the Baja California peninsula, Mexico.

**Significance/Conclusions:**

We have identified the set of conditions that lead to a persistent top predator hotspot, which increases our understanding of how highly migratory species exploit productive regions of the ocean. These results will aid in the development of spatially and environmentally explicit management strategies for marine species of conservation concern.

## Introduction

Highly migratory species are known to associate with dynamic and productive areas of the ocean such as coastal upwelling centers, fronts and eddies [1]. Many marine species have evolved migratory life history patterns to exploit biologically rich areas known as ‘hotspots’ as they move between foraging and breeding grounds [2]. Within many eastern boundary currents, primary production at localized upwelling centers sustains dense concentrations of prey species that, in turn, provide enhanced foraging opportunities for top predators [3]. Several studies have established relationships between predator distribution and physical or biological variables like bathymetry, sea-surface temperature (SST) or chlorophyll-*a* (e.g., [4]). However, most pelagic predators are several trophic levels removed from primary producers, such that relationships between abiotic factors that facilitate productivity and enhanced prey abundance may be indirect. Few studies have simultaneously measured the linkages between physical forcing, primary and secondary producers, and the pelagic predators that exploit them (but see [3]). Thus, while dynamic oceanic processes may indirectly attract predators, understanding the complete set of mechanisms leading to the formation of these highly productive hotspots requires knowledge of the spatio-temporal linkages that serve to concentrate prey within a patchy environment.

**Figure 1:**
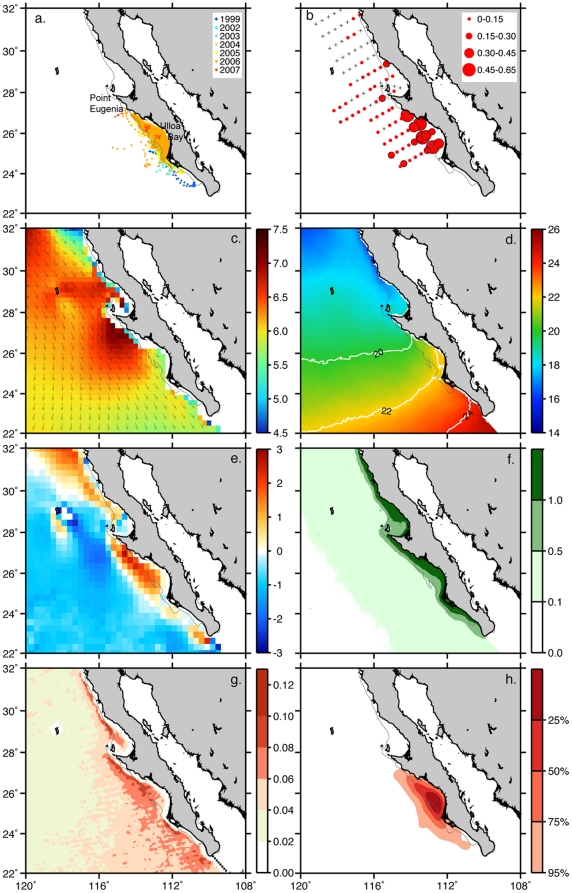
Spatio-temporal data averages off the Pacific coast of Baja California Sur, Mexico. Figure panels include: **(**A) Juvenile loggerhead turtle tracks (1999–2007; n = 30), (B) mean red crab abundance (log num m^−3^) at each IMECOCAL cruise station (2000–2008). Long-term averages of (C) surface winds (m s^−1^), (D) sea-surface temperature (°C), (E) vertical Ekman velocity (dm day^−1^), (F) chlorophyll-*a* (mg m^−3^), (G) frontal probability, (H) loggerhead turtle utilization distribution (%). Thin gray line represents the 200-m isobath.

The Pacific Ocean off the Baja California Peninsula (BCP), Mexico, has been identified as a hotspot for ecologically and economically important species inhabiting the California Current System (CCS), including tuna, sharks, sea turtles, seabirds, and whales [5], [6], [7], [8]. The pelagic red crab (*Pleuroncodes planipes*) is considered the principal intermediary in the energy flow from primary producers to a wide array of invertebrate and vertebrate predators, and probably serves as a major link that attracts a diverse assemblage of top predators to the BCP [9], [10]. Among these, juvenile loggerhead sea turtles (*Caretta caretta*) are unique in that they occur year-round, remaining tightly aggregated off the coast for decades (Figure 1A) before returning to their natal beaches off Japan to breed [11]. Optimal foraging theory would predict that juvenile loggerheads, which are not geographically constrained to centralized breeding grounds, should seek out productive areas that maximize growth during this stage [12]. Thus, the extended and localized presence of juvenile loggerheads off the BCP is indicative of a region of persistently favorable foraging conditions [13].

Red crabs occur at high densities in the waters off the BCP [14] where they are the primary prey of juvenile loggerheads [11], [15]. It has been hypothesized that strong ocean fronts concentrate red crabs, leading to increased abundance of foraging predators in this area [10], [16]. Previous studies have shown the correlation between red crab abundance and high chlorophyll-a concentrations off the BCP [15], [16], [17]. This study combines a suite of remotely sensed oceanographic measurements with long-term prey data sets and loggerhead satellite tracking to provide one of the first comprehensive assessments of the bottom-up creation of a top predator hotspot. These results offer valuable insight for the development of spatially based conservation strategies (e.g. marine protected areas and marine spatial planning) for top marine predators.

## Results

Long-term averages indicated that the predominant wind direction throughout the study area was from the northwest (Figure 1C). Winds were most intense around the Point Eugenia headland, with a long-term mean of 7 m s^−1^. Average SST showed a strong north-south gradient, increasing equatorward (Figure 1D). Elevated vertical Ekman transport in the water column, driven by positive wind-stress curl, was localized at three main locations along the coast (northern BCP, Ulloa Bay, and the southern tip of the BCP) (Figure 1E). Average chlorophyll-*a* concentrations>1 mg m^−3^ were found inshore along most of the coast (Figure 1F). On average, the highest probability of SST fronts extended as a band along the coast, narrowest off the northern BCP and widest south of Point Eugenia, especially offshore of Ulloa Bay (Figure 1G). The greatest mean abundance of adult pelagic red crabs was found within the shelf waters of Ulloa Bay, which is consistent with observations by [18], and extended offshore from this location (Figure 1B). Kernel density analysis of juvenile loggerhead turtles identified Ulloa Bay as the most highly utilized area off the entire BCP (Figure 1H).

**Figure 2:**
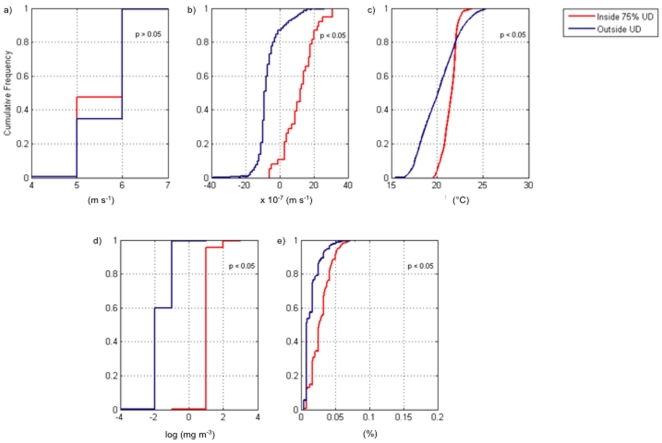
Empirical cumulative distribution plots of long-term conditions for environmental parameters found within high-use turtle habitat (red line) versus ambient environmental conditions (blue line). Figure panels include: (A) Surface winds, (B) vertical Ekman transport, (C) sea-surface temperature, (D) chlorophyll-*a*, and (E) frontal probability.

A comparison of the species-environment relationship between the high-use area vs. ambient conditions showed that all variables: sea surface temperature, chlorophyll-a, and frontal probability (Table 1) were statistically significant to turtle presence (p<0.05; K-S test), except for surface wind (Figure 2A). This might be due to the combination of a larger coastal land mask and spatial footprint (27.5 km) than most of the other variables, resulting in fewer data points for statistical analysis.

**Table 1:** Spatial and temporal resolution of satellite products.

Data set Name	Parameter	Sampling Interval	Spatial Footprint
AVHRR Pathfinder	Sea surface temperature	Daily	4.4 km
GOES	Frontal Probability Index	10-day composites	5.5 km
QuikSCAT	Wind fields	Daily	27.5 km
SeaWiFS	Chlorophyll-*a*	Daily	1.1 km

## Discussion

Several studies have examined the formation of upwelling “shadows” in the lee of coastal upwelling systems [19], [20]. Topographic irregularities shelter downstream embayments from intense wind, promoting steep thermal gradients and retentive circulation that entrains primary production and prey, and providing favorable foraging conditions to higher trophic levels [21], [22]. Our data indicate that the lee of Point Eugenia, Ulloa Bay, represents a unique assemblage of geopmorphological and physical oceanographic features, creating an upwelling shadow that serves to aggregate foraging loggerhead turtles (Figure 1A).

Along the coast, persistent positive wind-stress curl (Figure 1E), promotes upwelling and enhanced primary production (Figure 1F) by lifting the nutricline through Ekman suction, making light and key nutrients more readily available for phytoplankton. Within Ulloa Bay, the recirculation of water in the shadow provides relatively warmer SST (Figure 1D). The convergence of warm water with newly upwelled cold water results in frontal structures (Figure 1G). The combination of wind-stress curl and frontal structures maintain high densities of red crabs nearshore [23], providing enhanced high prey abundances along the shelf (Figure 1B).

As ectotherms, it is physiologically advantageous for sea turtles to reside in higher water temperatures (13–26°C) [24], which are favorable for locomotion, prey detection, and food assimilation [25]. However, colder waters are generally more productive. Thus, sea turtles are faced with balancing physiological advantages in warmer waters, against enhanced foraging in colder waters. Given that immature loggerheads exhibit a range of differential habitat strategies to match energetic costs with benefits [26], [27], their sustained presence within Ulloa Bay (Figure 2H) is indicative of a habitat preference that satisfies foraging and thermal requirements. The headlands of Point Eugenia provide a wind shelter for downstream Ulloa Bay, where warm water is entrained and adjacent to a region with enhanced upwelling. As a result, the upwelling shadow formed in Ulloa Bay acts as a productivity hotspot for loggerhead sea turtles, providing an area that is both thermally optimal for sea turtles, while juxtaposed to enhanced productivity and foraging opportunities. Indeed, spatial analysis shows that the environmental conditions within the core loggerhead hotspot (75% utilization density) are significantly different than the ambient conditions outside of this area (Figure 2).

There is a growing concern for marine species threatened by directed fishing [28], bycatch [6], and climate-driven shifts in suitable habitat [29]. Understanding the biophysical mechanisms that support productive habitats for foraging, shelter, and breeding is critical to marine spatial planners seeking to optimize conservation strategies [30]. This study highlights the ecophysiological importance of upwelling shadows by coupling satellite-based datasets to characterize the unique combinations of bottom-up processes in the formation of a productivity hotspot. Such approach may be broadly applicable to hotspots in upwelling shadows elsewhere, where knowledge of how these processes vary spatio-temporally will enable more effective marine conservation.

## Materials and Methods

### Data Tagging and Processing

Thirty juvenile loggerhead sea turtles were tracked from August 1999 to February 2007 (Figure 1A) by satellite platform transmitting terminals (PTT; Wildlife Computers, Redmond, Washington, USA). Turtles were caught by hand from small fishing boats and released within 18 h and 10 km from the capture location. Several turtles were retrieved from bottom-set longline or gillnet fisheries, and instrumented and released as stated above (see [6]). All necessary permits were obtained for the described field study.

Turtle positions were determined via the Argos satellite system [31], and only location classes identified as 1, 2, or 3 were included in the analyses. Raw positions with location classes Z, A, B and 0, as well as those with a maximum travel rate>5 km h^−1^ were filtered out. A land mask was applied to remove positions that occurred on land. Consecutive Argos location hits were interpolated every 12 h to reduce spatial autocorrelation (see [6], [32]). Satellite transmitter information for the 30 loggerhead sea turtles tracked off the Baja California Peninsula, Mexico (BCP), are listed in Table S1. Average track length was 139.6 ± 96.7 days.

### Prey Sampling

Hydrographic surveys were conducted by the Investigaciones Mexicanas de la Corriente de California (IMECOCAL) program (http://imecocal.cicese.mx). Surveys occurred seasonally (January-February, April, July, and September-October). Red crab samples were collected from oblique net tows [33] and recorded from 2000–2008.

### Remotely Sensed Oceanographic Data

Gridded digital bathymetry at 30 arc-seconds was extracted from the SRTM30_PLUS global database [34], and the 200-m isobath was extracted to identify the continental shelf waters. Remotely sensed oceanographic data include: surface wind speed, derived vertical Ekman velocity from wind-stress, sea surface temperature, chlorophyll*-a*, frontal probability (Table 1). Satellite oceanographic data were obtained for the study period from January 2000 - December 2007, except for frontal probability, a probability index that is calculated by the number of times a pixel is classified as a temperature front divided by the number of cloud free days for the given time period [35]. Frontal Probability was obtained from January 2001 - December 2007 due to data availability constraints.

Long-term averages were derived from binned arithmetic mean of monthly values, except for the chlorophyll-*a* average, in which case the geometric mean of monthly values was used due to the log-distributed nature of the dataset. Vertical Ekman transport was derived from wind-stress curl estimates [36].

### High-Use Area Analysis

Utilization distributions (UD) represent the probability of animal occurrence within a defined home range. Utilization distributions were determined using a Gaussian kernel density analysis of all interpolated positions [32], [37]. An index of turtle residence probability per unit area was computed by gridding the total number of turtle positions found within a 5-km^2^ cell. Each of these totals were then multiplied by the number of individuals present in that cell, thus weighting the cells more frequented by individuals for extended, high-use periods of time (see [13], [32]). Contours representing the percent area of habitat utilized were then generated from 1–99%. In order to determine the best UD contour line to use as the core area, we plotted the probability of use (%UD) by the percentage of home range use within the probability or greater [38]. The 75% contour had the maximum variation from random space use, and was the area most intensely used by juvenile loggerheads. For this reason, all areas within the 75% UD were considered to be core habitat. For display purposes, the UD contours at 25%, 50%, 75% and 95% were used (Figure 1H).

### Spatial analyses

In order to statistically quantify the spatial relationship between predators and oceanographic environment, we sampled oceanographic variables within the 1–75% UD contour intervals, and compared those values to “ambient” oceanographic conditions, which included all points between the 75% and 100% UD contours. We chose the area inside the 75% UD contour interval to represent high-use turtle habitat. We then plotted cumulative distribution functions for each environmental variable and ran a Kolmogorov-Smirnov test (K-S test) to determine statistical significance between the oceanographic conditions found within the core turtle habitat and outside, in the ambient environment.

## Supporting Information

Table S1 Satellite transmitter information for 30 loggerhead sea turtles (*Caretta caretta*) tracked off the BCP, Mexico.
(PDF)
