# Supplementary material for: The Making of a Productivity Hotspot in the Coastal Ocean
Source: PLoS One. 2011 Nov 21;6(11):e27874. doi: 10.1371/journal.pone.0027874 (PMC3221696; doi:10.1371/journal.pone.0027874)
Supplement: Table S1 — Satellite transmitter information for 30 loggerhead sea turtles (Caretta caretta) tracked off the BCP, Mexico. (PDF) [file pone.0027874.s001.pdf]

Table S1. Satellite transmitter information for 30 loggerhead sea turtles (*Caretta caretta*) tracked off the BCP, Mexico.

Tag ID	Deployment date	Last transmission	No. days monitored	Deployment latitude	Deployment longitude
17710	04-Aug-1999	27-Jan-2000	173	24.78	-112.10
21143	20-Jul-2002	22-Oct-2002	92	24.42	-112.21
22180	07-Aug-2002	19-Dec-2002	132	26.42	-113.00
22278	13-Sep-2002	19-Feb-2003	156	25.85	-112.91
21217	27-Aug-2002	11-Dec-2002	104	25.49	-112.68
20779	12-Aug-2003	03-Mar-2004	201	25.20	-112.38
20780	10-Aug-2003	10-Jan-2004	150	25.26	-112.28
42475	14-Jul-2004	03-Dec-2004	139	25.32	-112.45
42977	29-Jun-2005	08-Oct-2005	99	25.51	-112.25
42978	30-Jun-2005	06-Jan-2006	186	25.48	-112.47
42979	29-Jun-2005	10-Jul-2005	11	25.21	-112.36
42980	28-Jun-2005	20-Oct-2005	112	25.56	-112.35
42981	03-Jul-2005	07-Aug-2006	394	25.53	-112.35
42983	03-Jul-2005	28-Aug-2005	55	25.19	-112.13
60250	01-Sep-2005	25-May-2006	264	25.44	-112.33
29068	17-Jul-2006	16-Sep-2006	59	25.43	-112.36
42474	23-Aug-2006	27-Oct-2006	64	26.08	-112.46
57108	07-Jul-2006	01-Nov-2006	114	26.26	-112.48
57109	07-Jul-2006	05-Aug-2006	28	26.25	-112.48
57110	16-Jul-2006	29-Sep-2006	73	25.21	-112.16
57111	13-Aug-2006	07-Oct-2006	54	25.30	-112.30
57112	13-Aug-2006	10-Sep-2006	27	25.25	-112.26
60246	04-Aug-2006	04-Jan-2007	150	25.19	-112.13
60247	17-Mar-2006	30-Sep-2006	193	24.38	-112.72
64007	14-Jul-2006	08-Jan-2007	174	25.11	-112.56
64008	15-Jul-2006	17-Aug-2006	32	25.32	-112.20
64009	29-Aug-2006	24-Nov-2006	85	26.71	-113.60
64010	29-Aug-2006	08-Sep-2006	9	26.72	-113.63
68467	30-Aug-2006	02-Oct-2006	32	26.71	-113.57
68468	30-Aug-2006	01-Feb-2007	151	26.71	-113.57
